# Current diagnostic and therapeutic strategies for the management of lymphatic insufficiency in patients with hypoplastic left heart syndrome

**DOI:** 10.3389/fped.2023.1058567

**Published:** 2023-02-24

**Authors:** Christoph Bauer, Yoav Dori, Mario Scala, Andreas Tulzer, Gerald Tulzer

**Affiliations:** ^1^ Department of Paediatric Cardiology, Kepler University Hospital GmbH, Linz, Austria; ^2^ Johannes Kepler University Linz, Linz, Austria; ^3^ Department of Cardiology, Jill and Mark Fishman Center for Lymphatic Disorders, Children’s Hospital of Philadelphia, Philadelphia, PA, United States; ^4^ Central Radiology Institute, Kepler University Hospital GmbH, Linz, Austria

**Keywords:** lymphatic insufficiency, hypoplastic left heart syndrome, protein-losingenteropathy, plastic bronchitis, dynamic contrast magnetic resonance lymphangiography, innominate vein turn-down procedures, lymphatic interventional techniques, fontanoperation

## Abstract

Children with hypoplastic left heart syndrome share unique hemodynamic features that alter lymphatic integrity at all stages of palliation. Lymphatic congestion is almost universal in this patient group to some extent. It may lead to reversal of lymphatic flow, the development of abnormal lymphatic channels and ultimately decompression and loss of protein rich lymphatic fluid into extra lymphatic compartments in prone individuals. Some of the most devastating complications that are associated with single ventricle physiology, notably plastic bronchitis and protein losing enteropathy, have now been proven to be lymphatic in origin. Based on the new pathophysiologic concept new diagnostic and therapeutic strategies have recently been developed. Dynamic contrast magnetic resonance lymphangiography is now mainstay in diagnosis of lymphatic insufficiency and allows a thorough assessment of anatomy and function of the main lymphatic compartments through intranodal, intrahepatic and intramesenteric lymphatic imaging. Contrast enhanced ultrasound can evaluate thoracic duct patency and conventional fluoroscopic lymphangiography has been refined for evaluation of patients where magnetic resonance imaging cannot be performed. Novel lymphatic interventional techniques, such as thoracic duct embolization, selective lymphatic duct embolization and liver lymphatic embolization allow to seal abnormal lymphatic networks minimally invasive and have shown to resolve symptoms. Innominate vein turn-down procedures, whether surgical or interventional, have been designed to reduce lymphatic afterload and increase systemic preload effectively in the failing Fontan circulation. Outflow obstruction can now be managed with new microsurgical techniques that create lympho-venous anastomosis. Short term results for all of these new approaches are overall promising but evidence is sparse and long-term outcome still has to be defined. This review article aims to summarize current concepts of lymphatic flow disorders in single ventricle patients, discuss new emerging diagnostic and therapeutic strategies and point out lacks in evidence and needs for further research on this rapidly growing topic.

## Introduction

Lymphatic flow disorders in patients with hypoplastic left heart syndrome include some of the most devastating diseases associated with single ventricle physiology (1–3). Alterations in hemodynamics that are intrinsic to the Fontan circulation make individuals prone to lymphatic dysfunction and insufficiency with far reaching consequences for many organs (4). Lymphatic congestion is almost universal in this patient group to some extent. It may lead to reversal of lymphatic flow, the development of abnormal lymphatic channels and ultimately decompression and loss of protein rich lymphatic fluid into extra lymphatic compartments (5–7). In the thorax lymphatic insufficiency frequently manifests as postoperative chylothorax, plastic bronchitis or chylopericardium. In the abdomen protein losing enteropathy and ascites may be present. In some patients multicompartment lymphatic failure, the most severe form of lymphatic insufficiency, may develop over time (1, 8–10).

For decades, diagnostic and therapeutic strategies were limited. Advances both in diagnostic and therapeutic procedures over the past 10 years have now changed the perspective for these individuals vastly. The Fontan-population is constantly expanding in number and age (1). State of the art management of lymphatic insufficiency will become more and more important for the pediatric and adult cardiologist in the future. This article aims to provide current concepts of lymphatic flow disorders in single ventricle patients, discuss new emerging diagnostic and therapeutic strategies and point out lacks in evidence and needs for further research on this rapidly growing topic.

## Anatomy and physiology of the human lymphatic system

The lymphatic system is a discrete organ that is almost universal distributed in the body (11, 12). It consists of a network of thin, blind ending highly permeable lymphatic capillaries that drain excess interstitial fluid from the peripheral tissue into larger collecting lymphatic vessels and finally the thoracic duct or the right lymphatic duct before it enters the blood stream in the large veins of the upper body (13). We know that it is paramount for fluid balance, plays a key role in immune regulation and that it is important for transportation of micronutrients and intestinal absorption of long chain fatty acids (14–16). To fulfill all of its various tasks the structure of the lymphatic system is particular complex with a unique differentiation and distribution of the lymphatic vasculature and tissue in different organs (17). The anatomy is generally marked by a huge variability and plasticity and like blood vessels lymphatics are able to adopt to environmental tasks (12). Lymph propulsion is the result of intrinsic factors most important the contractions of smooth muscles of the larger collecting vessels and extrinsic factors. Flow direction is usually centripetal and is assured by valves that are interspersed in larger lymphatic vessels. Central lymphatic flow through the thoracic duct is approximately 2–3 liters a day, withdraw in can increase up to 30-fold under pathophysiologic conditions (18–20). About 80% of thoracic duct lymph flow comes from the abdominal viscera with the liver and the intestine being the single most important contributors (21). Regarding the lymphatics in patients after the Fontan operation there are hence three main components that are of special interest: the central lymphatic system including the thoracic duct, the liver lymphatic system and the mesenterial lymphatic system.

## The lymphatic system in adaptation to Fontan hemodynamics

The concept of lymphatic insufficiency in Fontan patients is currently not completely understood. The etiology is complex and most likely multifactorial (4). The Fontan operation was initially developed to reduce ventricular overload and cyanoses but came with the expense of an unphysiological pulmonary circulation. The lack of a sufficient sub-pulmonary pump inevitably leads to hemodynamic alterations in all individuals at all stages of palliation. Fontan physiology is best characterized by an elevated central venous pressure, loss of pulsatile pulmonary flow and a chronic reduced cardiac output. Once applied it poses an ongoing burden on almost all organs and ultimately leads to a variety of end organ consequences like liver disease, kidney disease or bone disease in many patients at some point in their life (22–24). As an integral part of the circulation the lymphatic system is particularly affected by the hemodynamic alterations.

**Figure 1:**
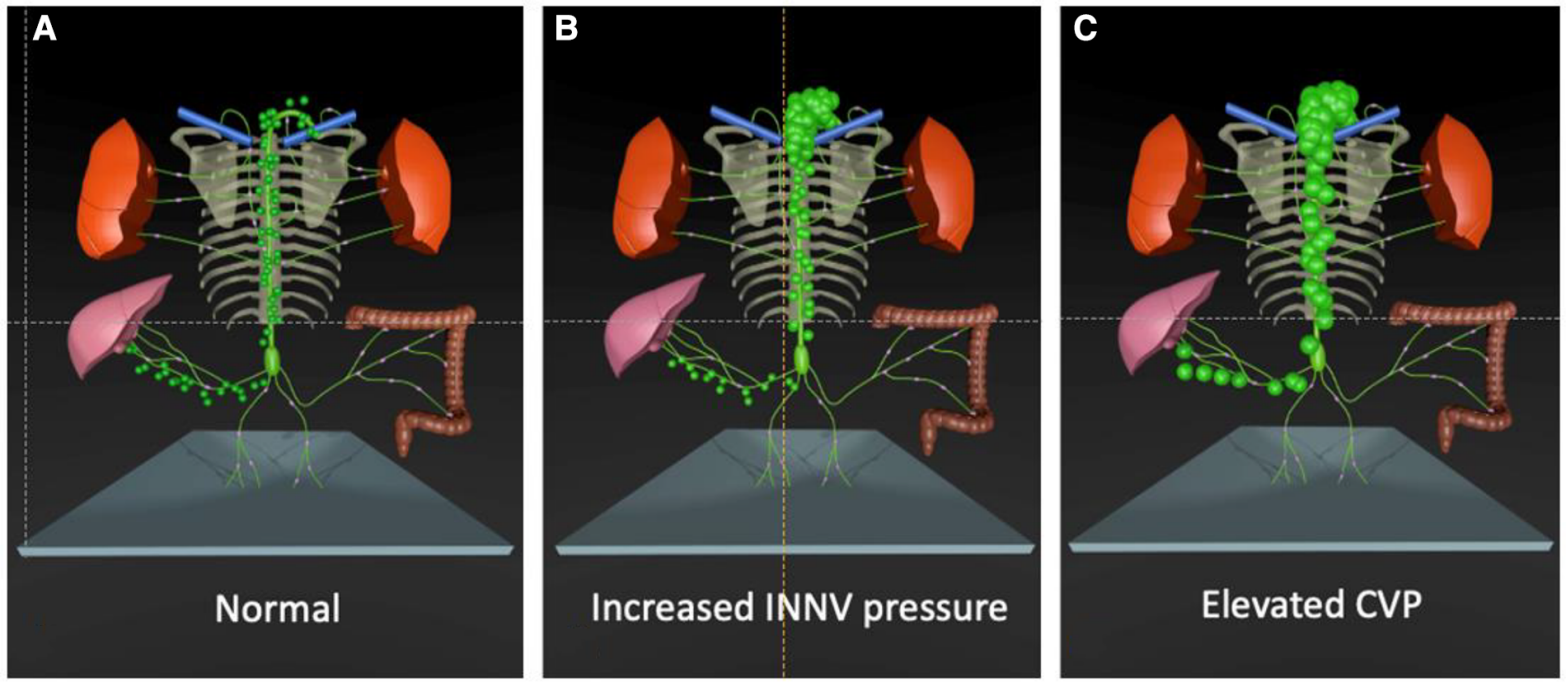
Effect of single ventricle palliation on lymphatic flow. (**A**) At birth lymphatic flow is normal. (**B**) After the Glenn operation the higher INNV pressure decreases lymphatic drainage capacity. (**C**) The elevated CVP after the Fontan operation additionally increases lymph production in the liver. INNV, innominate vein; CVP, central venous pressure.

The single most important pathophysiologic element is chronically elevated central venous pressure that is typically in the range of 12–15 mmHg in this cohort. It affects both lymph production and drainage capacity (Figure 1). According to the modified Frank Starling equation, that currently best describes the factors responsible for lymph production, augmented hydrostatic venous pressure leads to an increased efflux of fluid from the vasculature into the interstitial space. As a result, lymph production, especially in the liver and the intestine is stimulated (25) (Figure 1C).

The chronically high central venous pressure has further deteriorating effects on the liver parenchyma. It is well documented that subclinical liver fibrosis is present in almost all Fontan patients in adulthood (26). Some individuals further develop liver cirrhosis that may lead to portal hypertension. Previous studies have shown that the number of lymphatic vessels increases in fibrotic and cirrhotic livers and is positively correlated with the severity of liver disease. The molecular mechanisms behind these adaptations are largely unknown but an increase in VEGF, an inducer of lymph angiogenesis, may play a role. Apart from the increased lymph production the ability to absorb interstitial fluid is reduced in cirrhotic livers. In addition, the decreased oncotic pressure caused by liver failure or loss of plasma proteins in protein losing enteropathy potentially alters reabsorption of interstitial fluid and further stimulate lymph production. But this mechanism seems not to play a major role (6). The elevated innominate vein pressure also increases lymphatic afterload at the lympho-venous junction and cause lymphatic congestion in almost all Fontan patients (Figure 1B). In order to drain into the venous system, lymph from the thoracic duct has to overcome a similar or even higher pressure than it is produced (5, 22). A well-functioning lymphatic pump is especially important in that setting. However, there is increasingly evidence that drainage capacity is diminished in Fontan patients (27). The thoracic duct diameter has been found to be enlarged in Fontan patients especially when there is lymphatic failure (28). Lymphangion dilation may also have effect on valve function and contractility (6). Evidence of lymphatic dysfunction in Fontan patients has recently been demonstrated by near infrared fluoroscopic imaging showing diminished lymphatic pumping, decreased lymphatic pumping pressure and a higher contraction frequency in the lower extremities (22). At least elevated central venous pressure may also alter lympho-venous valve function, that it is mediated by venous wall tension (29).

Although elevated central venous pressure is the driving force for lymphatic insufficiency in Fontan patients there is no direct correlation of the magnitude of central venous pressure to the occurrence of lymphatic disease. It is well known that there are some patients with severely elevated central venous pressure that do not have lymphatic problems while others with relatively low central venous pressure may have severe lymphatic issues (19). It is now thought that a congenital or genetic susceptibility with possibly anatomic variations that bring lymphatic channels in proximity to serous surfaces where they can decompress, may be an important factor. Besides altered mesenteric vascular resistance, inflammation has also been implicated to possibly play a significant role in several lymphatic related diseases (30, 31).

## Lymphatic flow disorders in single ventricle patients

Lymphatic flow disorders manifest at all stages of single ventricle palliation, most often after the Glenn operation the Fontan completion or later in life, when the burdened lymphatic system finally decompresses into extra lymphatic compartments causing effusions or leading to tissue edema and organ dysfunction (1). In some patients multiple compartments may be involved.

**Flow disorders of the Thorax:**

**Figure 2:**
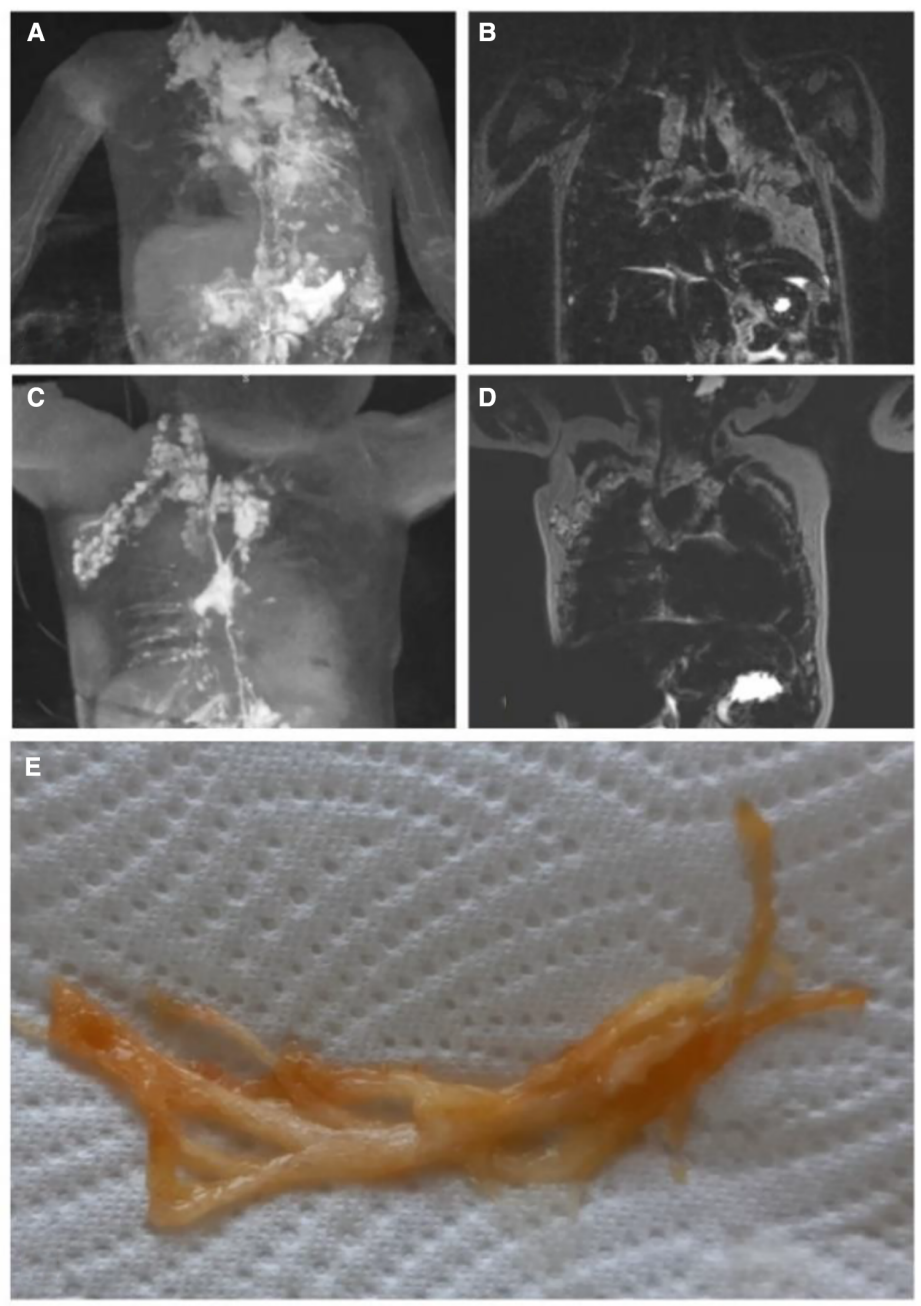
Pulmonary lymphatic perfusion syndrome. (**A**) Coronal projection of intranodal, intrahepatic and intramesenteric dynamic contrast magnetic resonance lymphangiography and (**B**) corresponding MIP T2 weighted lymphatic imaging demonstrating retrograde perfusion of the mediastinal, peribranchial- and pulmonary interstitial-lymphatic networks in a 3.2 year old patient with occluded thoracic outlet (type 4). (**C**) Coronal projection of intranodal dynamic contrast magnetic resonance lymphangiography and (**D**) corresponding MIP T2 weighted lymphatic imaging demonstrating a double thoracic duct in a 2.5 year old patient with the left duct supplying the lungs (type 3). (**E**) Typical airway cast in PB. MIP, maximal intensity projection; PB, plastic bronchitis.

There are three main types of lymphatic flow abnormalities in the chest that can frequently be seen in single ventricle patients. These include lymphatic pleural effusions, lymphatic pericardial effusions and plastic bronchitis (9, 32, 33). The etiology of all of them is similar and almost always due to retrograde perfusion of the mediastinal, peribranchial- and pulmonary interstitial-lymphatic networks. This flow pattern has previously been termed pulmonary lymphatic perfusion syndrome (PLPS) (Figures 2A–D) (9).

Prolonged pleural effusion is common, especially after the creation of a bidirectional cavopulmonary connection or total cavopulmonary connection in single ventricle patients and affects up to 12,6% and 37%, respectively (34). Prolonged mechanical ventilation after surgery may worsen lymphatic dysfunction and has therefore to be avoided (35).

**Figure 3:**
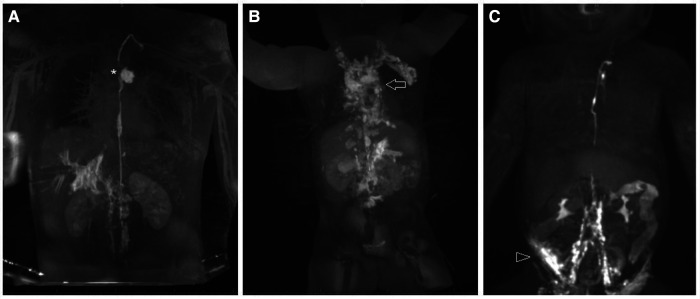
Different etiologies of chylothorax. Coronal projection of dynamic contrast magnetic resonance lymphangiography in a a 15 year old patient with (**A**) traumatic leak (asterixis), in a 7 year old patient with (**B**) pulmonary lymphatic perfusion syndrome (arrow) and (**C**) a central lymphatic flow disorder showing dermal backflow (arrowhead) in a 2 months old patient.

Chylothorax and chylopericardium is seen when lymph originating from the mesenteries drains to the pleural or pericardial space (8, 9). The diagnosis is made when the effusion consists of triglyceride levels greater than 110 mg/dl in a patient on full fat diet, has high lymphocyte percentage (80%–100%) and chylomicrons are present (25). It is most commonly seen after Fontan completion and may affect up to 24% of patients but can also occur in neonates (36–38). In a small study of 18 patients suffering from chylothorax after congenital heart surgery three distinct etiologies could be described based on dynamic contrast magnetic resonance lymphangiography and inguinal lymphangiography (Figure 3). Two patients had a traumatic leak (Figure 3A), fourteen a pulmonary lymphatic perfusion syndrome (Figure 3B) and nine a central lymphatic flow disorder (Figure 3C) (9).

Plastic bronchitis is characterized by the formation of rubbery like casts (Figure 3E) that obstruct the airways and may lead to life threatening asphyxia. 4%–14% of Fontan patients may be affected (29, 39). Mortality was historically high but could be improved in the current era. Patients are usually presenting with chronic cough, wheezing and dyspnea, symptoms that are similar to other common airway disease. In addition, plastic bronchitis symptoms are often triggered or diminished by infections. It is therefore not rare that they are first misdiagnosed as asthma or pneumonia, especially in children that are often swallowing the casts (1). The diagnosis can be confirmed with bronchoscopy in such cases. A high suspicion is necessary in order not to delay diagnosis.

**Flow disorders in the Abdomen:**

Lymphatic abnormalities in the abdomen are more heterogenous and complex than in the thorax and somehow less well defined. They include protein losing enteropathy as well as chylous and non-chylous ascites.

**Figure 4:**
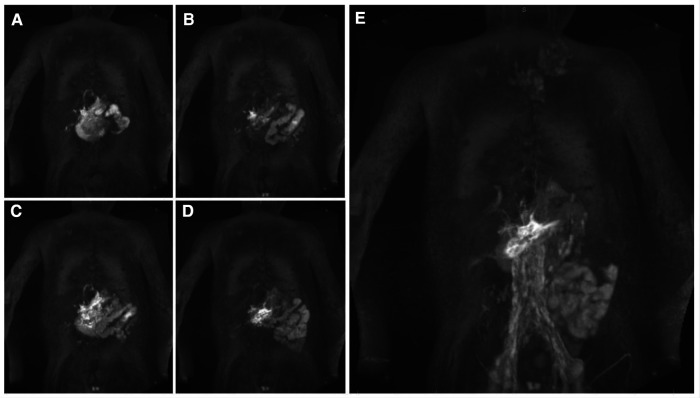
DCMRL sequence of all major lymphatic streams in a 7 year old patient with PLE. Coronal projection of (**A,B**) intrahepatic DCMRL, (**C,D**) intramesenteric DCMRL, and (**E**) intranodal DCMRL revealing leakage into the duodenum. DCMRL, dynamic contrast magnetic lymphangiography;.

Protein losing enteropathy is the most common lymphatic flow disorder in the abdomen and affects 5%–12% (40, 41) of Fontan patients. Intrahepathic and intramesenteric dynamic contrast magnetic resonance lymphangiography recently demonstrated abnormal increased lymphatic flow from the liver lymphatics (Figures 4A,B) or to a lesser extend from the mesenterial lymphatics (Figures 4C,D) to the duodenum in these patients (42, 43). Duodenal wall edema, inflammation and lymphangiectasia is commonly seen on endoscopy and even holes formed in the duodenal wall have been demonstrated in later stages in these patients (25). In patients with protein losing enteropathy a variable degree of hypoalbuminemia, hypogammaglobulinemia and lymphopenia is usually seen. They are commonly presenting with peripheral edema, ascites, pleural or pericardial effusions and abdominal complaints like diarrhea, abdominal bloating and pain. Malabsorption in these patients can lead to a chronic catabolic status with severe malnutrition and weight loss. The severity of protein losing enteropathy symptoms are widely variable and range from subclinical presentations to multiorgan involvement. Like in plastic bronchitis symptoms can be transient and often worsen during states of inflammation and over time (1, 40, 44). The diagnosis is based on clinical presentation, confirmation of enteric protein loss and the exclusion of other causes for hypoproteinemia (45). Despite improvement in diagnostic and therapeutic strategies in the last years, management of protein losing enteropathy is a challenge and is still associated with increased morbidity and mortality (4).

Chylous and non-chylous ascites is commonly seen in Fontan patients especially with a failing Fontan circulation and may affect up to 30% of adults. Truly chylous ascites is rare and can result from traumatic lymphatic leakage. In non-chylous ascites the etiology is due to mesenteric lymphatic congestion, but portal hypertension in Fontan associated liver disease, hypoalbuminemia, renal dysfunction and congestive heart failure with low cardiac output may play a role too (25).

**Multicompartment lymphatic failure:**

**Figure 5:**
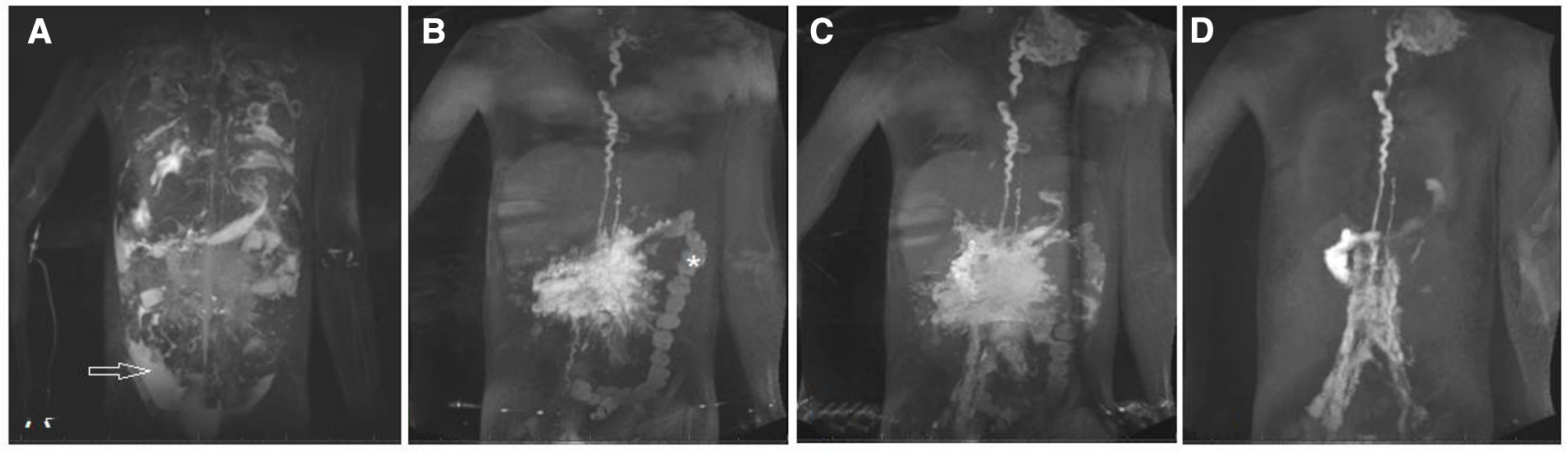
MIP coronal projection of (**A**) T2 imaging and (**B–D**) DCMRL in a 17.9 year old patient with multicompartment lymphatic failure including ascites (arrow), PLE (asterixis). DCMRL, dynamic contrast magnetic resonance lymphangiography; PLE, protein losing enteropathy; DCMRL, dynamic contrast magnetic resonance lymphangiography.

Multicompartment lymphatic failure is the most severe manifestation of lymphatic insufficiency in Fontan patients. By definition it involves a combination of lymphatic perfusion abnormalities in at least two compartments including the thorax, the abdomen and the soft tissue. It can manifest in both neonates and older patients and is difficult to manage (Figure 5) (46). In young patients the term central lymphatic flow disorder describes multicompartment failure that is a combination of abnormal central lymphatic flow, effusions in more than one compartment and the presence of dermal backflow through lymphatic collaterals in the abdominal wall (Figure 3C). It affects infants aged less than 1 year and has a particular poor prognosis (9). In older infants and adults, multicompartment lymphatic failure is most often a combination of thoracic lymphatic abnormalities like chylothorax chylopericardium or plastic bronchitis with protein losing enteropathy and or ascites.

## Novel imaging modalities of the lymphatic system

The development of innovative minimally invasive lymphatic imaging techniques during the last decades were pivotal for the investigation of current pathophysiologic concepts of lymphatic flow disorders and the introduction of new lymphatic interventional procedures. The term lymphatic imaging nowadays summarizes a spectrum of novel imaging modalities that have been established recently to visualize the lymphatic system. Historically pedal lymphangiography and lymphoscintigraphy have been used but due to their poor special and temporal resolution these modalities have little to no role in the current era (47). T2-magnetic resonance lymphangiography and dynamic contrast magnetic resonance lymphography are now mainstay in the diagnosis of lymphatic flow disorders (48, 49). Because of the huge variability in lymphatic anatomy and abnormal flow pattern seen in lymphatic disease a systematic assessment of every patient with suspected lymphatic disease is crucial before initiation of therapy.

Non contrast heavily T2 weighted magnetic resonance lymphangiography imaging can easily be implemented during routine cardiac magnetic resonance imaging or as part of a thorough workup. It depicts areas of slow-moving non-bloody fluids resembling lymphatic tissue with high spatial resolution and serves as a screening tool for abnormal lymphatic distribution in the neck, the thorax and abdomen but lacks dynamic information. It can be done in any pediatric patient who is suitable for MRI.

**Figure 6:**
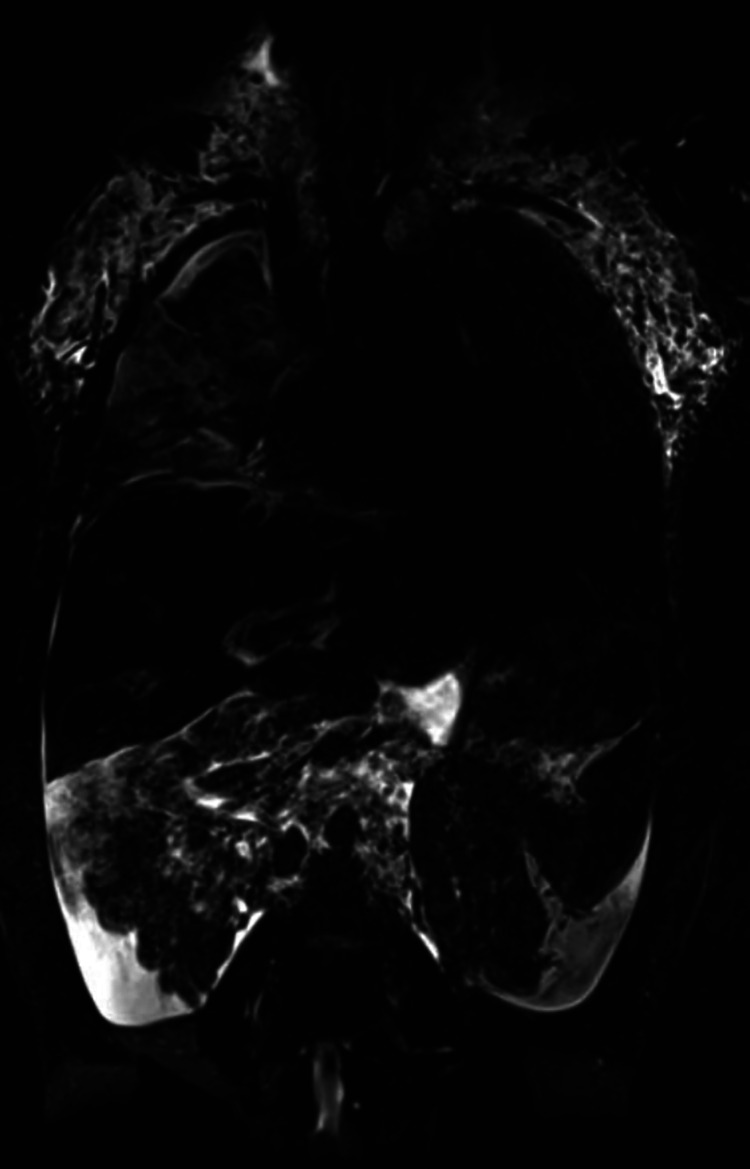
Native T2 weighted lymphangiography coronal projection depicting type 4 lymphatic abnormality with bilateral supraclavicular lymphatic abnormality and lymphatic networks extending into the lung parenchyma in a 13 year old patient.

Based on this imaging modality Biko et al. have published four types of lymphatic abnormality in patients after superior cavo pulmonary connection. In this study infants with higher degree of neck and thoracic lymphatic abnormalities were more likely to have failure of Fontan completion and a longer postoperative stay. Most notably a need for cardiac transplantation and death only occurred in infants with involvement of the lung parenchyma (type 4) (Figure 6). This imaging should therefore be used as a screening tool in all patients prior to Fontan completion to assess postoperative risk (48).

### Dynamic contrast-enhanced magnetic resonance lymphangiography

For functional assessment of lymphatic flow, dynamic contrast MR lymphangiography has been developed (DCMRL) (47).

The principle of all dynamic contrast magnetic resonance lymphangiography categories is that Gadolinium is applied into distinct lymphatic compartments and magnetic resonance images are gathered in a time resolved manner. With this approach flow dissemination and direction can be visualized. As contrast can only be seen in the parts of the lymphatic system that are in the contrast pathway it is necessary to use different access points depending on the clinical presentation. The three main access points are intranodal for the central lymphatics, intrahepatic for hepato-systemic lymphatics and intramesenteric whose role is not well-defined yet. It can even be done in very small infants that weight approximately 3 kg (Figures 4A–E) (42, 49, 50). In patients where multiple compartments are involved all three access points can be investigated at the same time. In that case the contrast medium that is applied can be diluted.

Intranodal dynamic contrast-enhanced magnetic resonance lymphangiography was first developed and involves the cannulation of lymph nodes in the groin (51). Usually a 25-gauge spinal needle is advanced under ultrasound guidance into the hilum of superficial inguinal lymph nodes on both sides. Good position is then confirmed either by injecting an iodine contrast agent under fluoroscopy or a small amount of natriumchloride under ultrasound guidance which normally leads to a dilatation of the lymph node. After securing the needles with Tegaderm^©^ the patient is transferred into the magnetic resonance suite for imaging. At first non-contrast native T1 and T2 weighted sequences are gathered mostly in 3D followed by an injection of contrast into the lymph nodes. We usually administer a total of 0.2 ml per kg of bodyweight of macrocyclic Gadolinium divided to the different injection sites administered by hand injection at a slow rate. Dynamic sequences depend on the MRI manufacturer but are available on all platforms. Timing depends on dynamics of the lymphatic flow but normally for intranodal injection 10–15 min of dynamic imaging followed by several high resolution contrast enhanced sequences is sufficient.

Usually, the lymph flow first propels contrast to the retroperitoneal lymphatics, then the cisterna chyli and the thoracic duct. In Fontan patients this method is most useful to detect retrograde flow to the lungs, the pleural space or the pericardial sac (Figure 2D). In protein losing enteropathy patients lymphatic fistulas from the central lymphatics to the duodenum are the exception but may be seen.

Based on intranodal dynamic contrast-enhanced magnetic resonance lymphangiography Dori et al.l. described six patterns of abnormal pulmonary lymphatic perfusion that can be seen in most patients with plastic bronchitis (52). In this study most patients had a patent thoracic duct with retrograde flow into multiple branches.

Intrahepatic dynamic contrast magnetic resonance lymphangiography is able to detect hepatic lymphatic connections and flow patterns that may be important for the treatment of ascites, chylothorax, plastic bronchitis and protein losing enteropathy (42, 43). Prior to magnetic resonance imaging, lymphatic access is usually gained in the cardiac catheterization laboratory using a bimodal approach. At first a 25-gauge spinal needle is positioned next to a branch of the portal vein under ultrasound guidance. Afterwards needle position is adjusted under fluoroscopy while applying iodine contrast until liver lymphatics are clearly seen and the needle can be secured with Tegaderm^©^. Careful transfer of the patient to the magnetic resonance imaging table and an akinetic transport into the magnetic resonance imaging suite is essential and may pose a challenge when a hybrid catheterization-magnetic resonance imaging laboratory is not available. However, our experience is that intrahepatic needle position is quite stable despite the common breathing movements.

With intrahepatic dynamic contrast magnetic resonance lymphangiography four abnormal categories of liver lymphatic flow may be seen with high correlation to presenting disease. Hepatoduodenal connections are frequently seen in patients with protein losing enteropathy and are the major feeding vessels in this patient group (Figure 4A). Hepatopulmonary flow patterns are rarer but are seen in patients with chylothorax and plastic bronchitis. Patients with ascites are more likely to show hepatoperitoneal connections. Only retrograde perfusion of the mesenteric lymphatics from the liver intrahepatic is unspecific and may be present in all disease categories (42, 50).

In another small study hepatoduodenal flow led to an enhancement of the duodenal wall during intrahepatic dynamic contrast magnetic resonance lymphangiography and leakage into the lumen in most protein losing enteropathy cases. However, enhancement of the duodenal wall was also seen in a small proportion of non-protein losing enteropathy patients. This finding might represent pre-symptomatic protein losing enteropathy or a cohort at risk for protein losing enteropathy and has to be further investigated. Intrahepatic dynamic contrast magnetic resonance lymphangiography may therefore have a role in screening of patients at risk or pre-operative risk-assessment for developing protein losing enteropathy in the future (42).

Intramesenteric dynamic contrast-enhanced magnetic resonance lymphangiography has been introduced most recently and may be particularly valuable in patients with ascites and protein losing enteropathy (Figures 4C,D). Lymphatic access is thereby gained similar to intrahepatic dynamic contrast magnetic resonance lymphangiography using a bimodal approach. Under ultrasound guidance mesenteric lymphatic ducts or lymph nodes in the anterior portion of the small intestine are targeted with a 25-gauge spinal needle. Accurate position is confirmed with fluoroscopy before a dynamic time resolved magnetic resonance lymphography is performed. Although it is more demanding and needle position is less stable than in intrahepatic and intranodal dynamic contrast magnetic resonance lymphangiography a success rate of 93% has been reported in a preliminary retrospective study of 15 preselected patients. In this analysis intramesenteric dynamic contrast magnetic resonance lymphangiography was most accurate in detecting peritoneal leaks and showed a duodenal leak not seen on the intrahepatic approach. However, as evidence is limited to the above-mentioned study safety and utility has to be confirmed in further studies (50).

### Ultrasound

Ultrasound is crucial in guiding intranodal, intrahepatic and intramesenteric lymphatic access. In addition, the cervical part of the thoracic duct, including the thoracic duct outlet and the lympho-venous valve can be imaged with gray scale high resolution ultrasound. It allows the assessment of local anatomy, imaging of valvular function and even detection of chyle flow in the terminal part (53, 54). The use of microbubble ultrasound contrast agents has recently expanded the diagnostic potential of ultrasound in lymphatic disease. It is well established in adults and has now gained attention in children in various diagnostics and invasive procedures (55, 56). Sulfur hexafluoride lipid-type A microspheres are most often used and have been shown to be save in children. It is now applied in a variety of settings including the evaluation of pathology in the liver, lung, spleen, brain, pancreas, bowel, kidney and the female pelvis (56).

In lymphatic disease contrast enhanced ultrasound has successfully been used to assess lymph nodes and superficial lymphatic networks in the extremities (57). Injection of contrast inside inguinal lymph nodes is an effective technique to confirm the position of the needles before intranodal dynamic contrast magnetic resonance lymphangiography when fluoroscopy is not available (58). During intranodal or intrahepatic dynamic contrast magnetic resonance lymphangiography thoracic duct outlet cannot be directly visualized and therefore fails to accurately demonstrating thoracic duct patency. Contrast enhanced ultrasound is a simple, save method that allows direct visualization of lymphatic flow into the venous system to confirm patency and may become a routine procedure in the future before or after dynamic contrast magnetic resonance lymphangiography (54). This is particularly important in patients where obstruction of the thoracic duct outlet is suspected as the success of innominate vein turn down procedures or lymphovenous anastomosis is highly dependent on unobstructed flow in these patients.

Ultrasound has many advantages in contrast to other imaging modalities that are particularly important in children. It is fast, non-invasive, avoids ionizing radiation and sedation, provides excellent spatial resolution and offer the advantage of real time imaging at bedside (59). Especially in small children acoustic windows are much better and may widen application of this technique in the future like preoperative screening for lymphatic susceptibility or helping in guiding during invasive procedures.

### Conventional fluoroscopic lymphangiography

Conventional fluoroscopic lymphangiography can be used in addition to dynamic contrast magnetic resonance lymphangiography or solely to evaluate the lymphatic system in infants with contraindication to magnetic resonance imaging (i.e., pacemaker dependence). After gaining lymphatic access as described before, iodinated water-soluble contrast agents or lipiodol is administered. Compared to dynamic contrast magnetic resonance lymphangiography opacification of the central lymphatic system is usually profoundly poorer especially with iodinated agents. Lipiodol provides somewhat better contrast but has been associated with some severe complication including cerebral stroke (60–62). It must therefore be used with caution in patients who have potentially right to left shunt. In our institution screening for shunts before lipiodol administration and before all interventions is therefore routinely performed. Veno-venous collateral vessels and lympho-pulmonary-vein connections, can be closed before, and an open fenestration may be temporary closed during the procedures. However, blocking a fenestration with a balloon may pose the risk of thrombus formation and subsequent embolism, especially during intervention where anticoagulation is prohibited.

During lymphatic procedures where the thoracic duct is accessed and contrast can be directly applied through a microcatheter, conventional fluoroscopy can add valuable information about anatomy and may even reveal small fistulas not seen on dynamic contrast magnetic resonance lymphangiography.

## Management of lymphatic insufficiency

**Figure 7:**
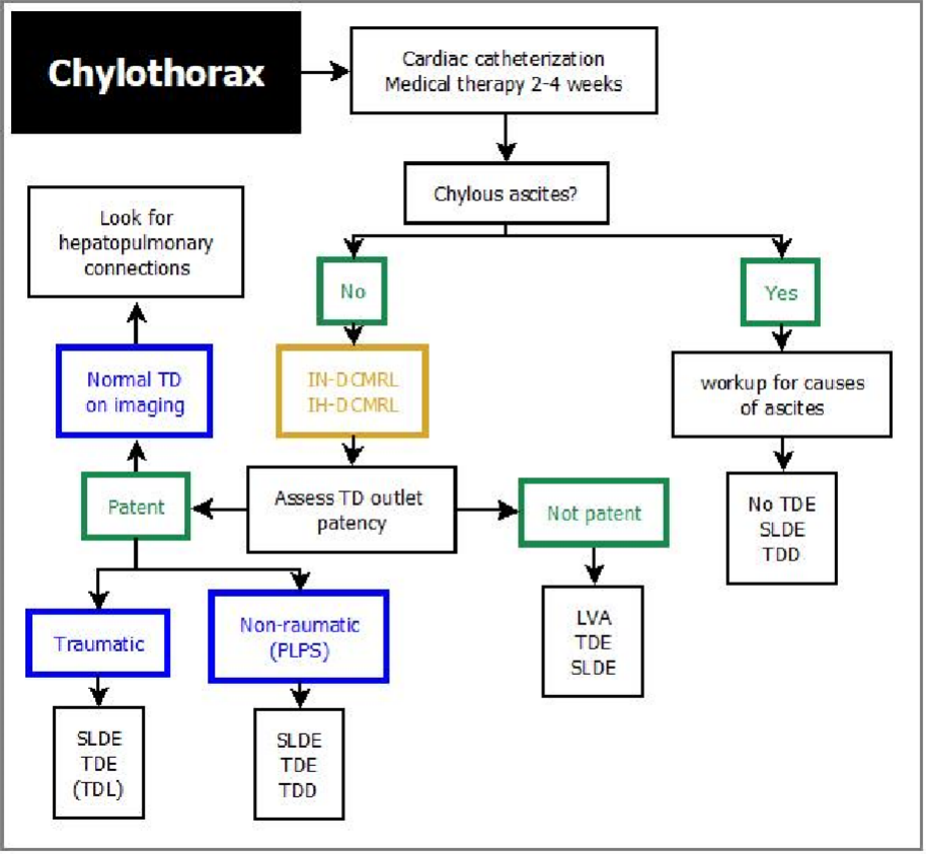
Treatment algorithm for chylothorax. DCMRL, dynamic contrast magnetic resonance lymphangiography; IH, intrahepathic; IN, intranodal; LVA, lymphovenous anastomosis; TDE, thoracic duct embolization; SLDE, selective lymphatic duct embolization; TD, thoracic duct; TDD, thoracic duct decompression; TDL, thoracic duct ligation; LVA, lympho-venous anastomosis.

**Figure 8:**
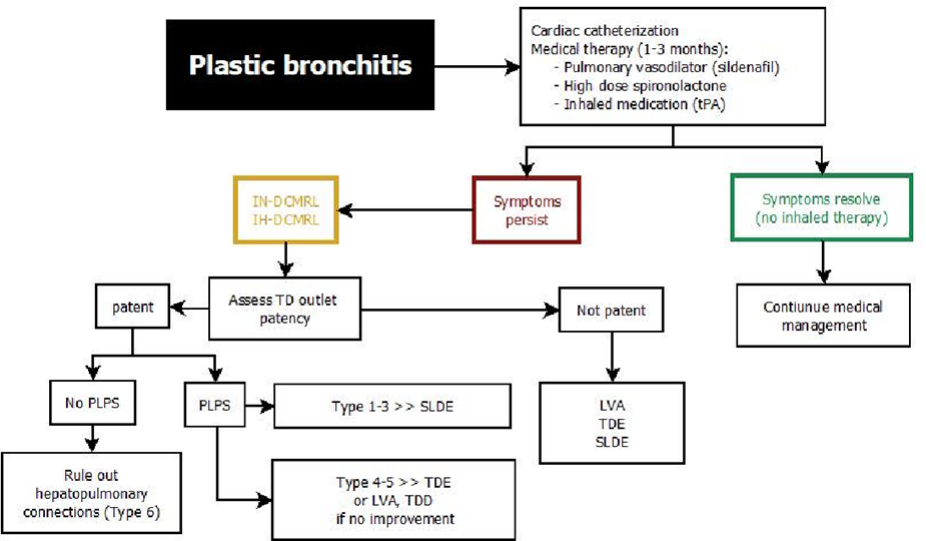
Treatment algorithm for plastic bronchitis. DCMRL, dynamic contrast magnetic resonance lymphangiography; IH, intrahepathic; IN, intranodal; LVA, lymphovenous anastomosis; PLPS, pulmonary lymphatic perfusion syndrome; SLDE, selective lymphatic duct embolization; TDE, thoracic duct embolization; TDD, thoracic duct decompression; LVA, lympho-venous anastomosis.

**Figure 9:**
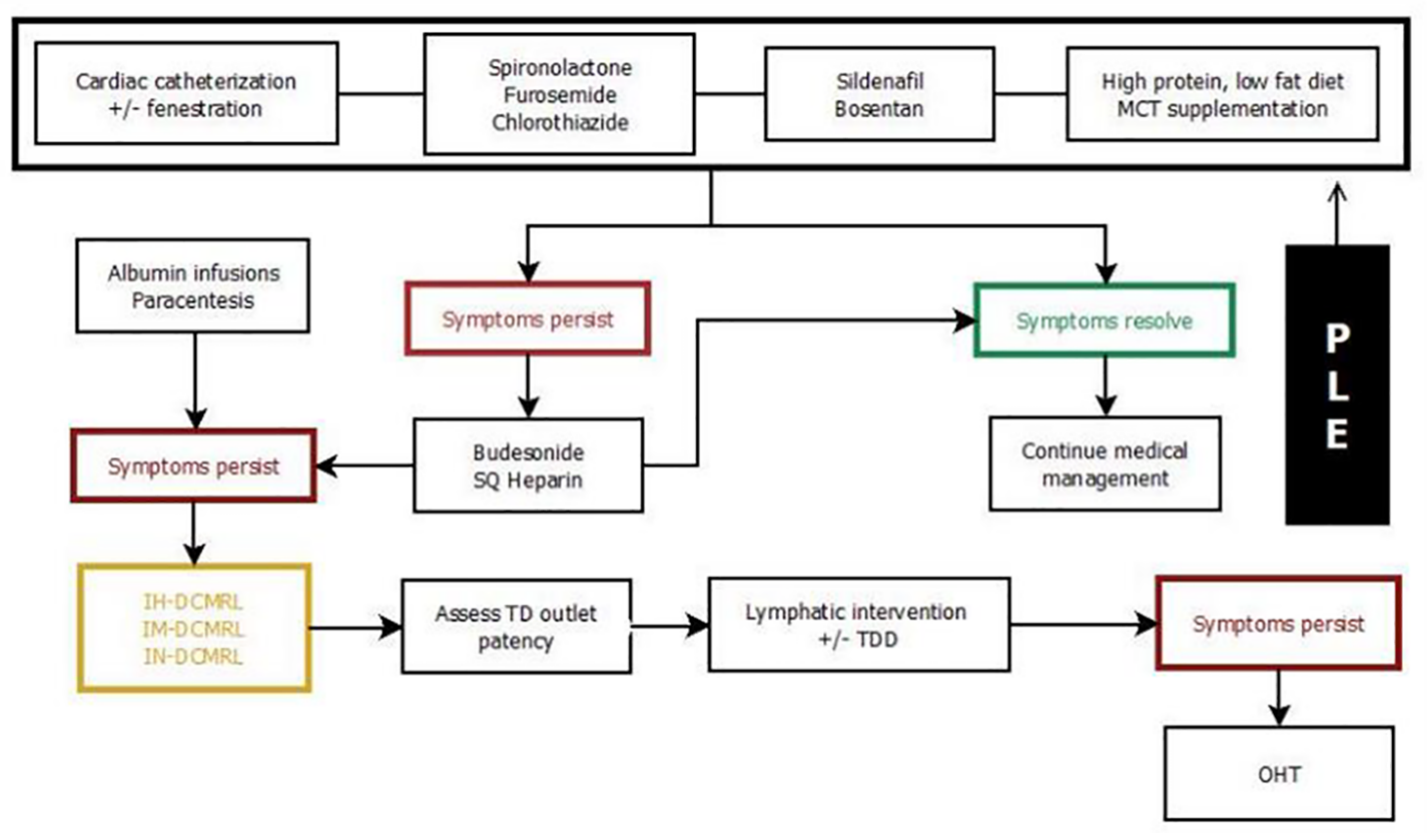
Treatment algorithm for protein losing enteropathy. DCMRL, dynamic contrast magnetic resonance lymphangiography; IH, intrahepathic; IN, intranodal; IM, intramesenteric; TDD, thoracic duct decompression; MCT, medium-chain triglyceride; PLE, protein losing enteropathy.

**Figure 10:**
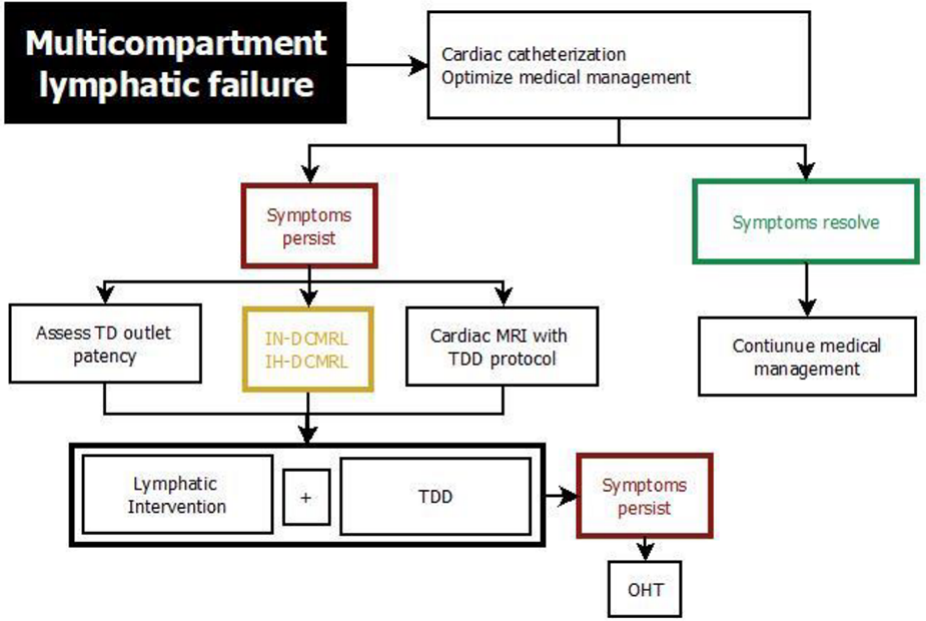
Treatment algorithm for multicompartment lymphatic failure. DCMRL, dynamic contrast magnetic resonance lymphangiography; IH, intrahepathic; IN, intranodal; IM, intramesenteric; TD, thoracic duct; MCT, medium-chain triglyceride; OHT, orthotopic heart transplant.

Modern management of lymphatic insufficiency involves medical, interventional and surgical strategies and is multidisciplinary (1, 4, 63). Treatment algorithms have recently been proposed for most lymphatic flow disorders and may help to establish a more uniform diagnostic and therapeutic approach throughout centers (Figures 7–10) (25, 46). Initially a thorough assessment of each individual is necessary to evaluate Fontan pathways, underlaying pathophysiology and to rule out differential diagnosis. All patients with suspected lymphatic abnormalities should therefore undergo cardiac catheterization to exclude systemic obstruction and to assess atrioventricular valve competence, pulmonary anatomy and pulmonary vascular resistance. In our institution a Holter electrocardiogram is conducted to search for conduction system abnormalities that can be addressed. In patients with protein losing enteropathy gastroscopy and duodenoscopy can be useful to exclude differential diagnosis or concomitant disease like cytomegalovirus infection that may warrant further treatment (64).

### Conservative management

Medical therapy is often symptomatic and supportive although there are medications that are thought to improve altered hemodynamics or have effects on lymph production. Evidence for all these drugs is generally low and often limited to case reports, case series or expert opinions (1).

In most centers a combination of diuretics and pulmonary vasodilators is used as a basic therapy to treat lymphatic insufficiency in most Fontan patients. Diuretics, like furosemide are widely subscribed to reduce venous and lymphatic congestion and to treat edemas and effusions. Aldosterone inhibitors have similar effects and may reduce inflammation and improve ventricular function. Pulmonary vasodilators (i.e., PDE-5 inhibitors, endothelin-1 receptor inhibitors) are aimed to reduce pulmonary resistance and thereby reduce lymphatic afterload and congestion and can be beneficial in most individuals (1, 46, 65).

In protein losing enteropathy patients serial albumin replacements sometimes are required to increase oncotic pressure and alleviate edemas and effusions. Intravenous immunoglobulin can aid in patients with opportunistic infections and may be administered in addition to replacing chronically low serum immunoglobulin that are commonly observed in this patient group. Interestingly, despite the alterations in immunoglobulin levels and lymphocyte counts Fontan patients usually are not prone to severe infections (66–68). Oral controlled-release budesonide has been shown to increase albumin levels in some case series (69, 70). However, despite the assumption that budesonide has a high first pass effect in the liver, side effects are frequently observed with this medication. In addition, it seems that the effect is more pronounced in young infants and decreases with disease progression. Some patients do not benefit at all and therefore corticosteroids should be used with caution and its benefits should frequently be reevaluated. In some centers unfractionated heparin and somatostatin have successfully been used to treat protein losing enteropathy. It has been shown that enterocytes in Fontan patients with protein losing enteropathy lack heparan sulfate and heparin therefore is thought to reduce membrane permeability and have a positive effect on intestinal inflammation in these individuals (40, 71, 72).

In patients suffering from plastic bronchitis a number of treatments to relive airway obstruction and anti-inflammatory agents have been used. Bronchodilators and chest physiotherapy can be started in all individuals that are regularly producing casts (1). An acute exacerbation of plastic bronchitis is often treated with inhaled mucolytics like dornase-α or N-acetylcysteine and inhaled corticosteroids. Nebulized tissue plasminogen activator has been shown to reduce cast weight in spontaneously expectorated plastic bronchitis casts and is used to clear obstructive airway casts in acutely symptomatic individuals (73, 74). In any individual with acute airway obstruction bronchoalveolar lavage and removal of airway casts should not be delayed and is lifesaving.

Dietary modifications are aimed to reduce lymph production. Total parenteral nutrition can be useful in the setting of postoperative chylothorax. A high-protein, low-fat diet with a greater-than-normal proportion of medium-chain triglycerides, may be beneficial in some protein losing enteropathy and plastic bronchitis patients but malnourishment must be avoided (65, 75). Fontan patients especially with protein losing enteropathy are at increased risk for iron deficiency, hypocalcemia and vitamin deficiency that may have to be substituted frequently (76–78).

### Surgical and interventional strategies

**Figure 11:**
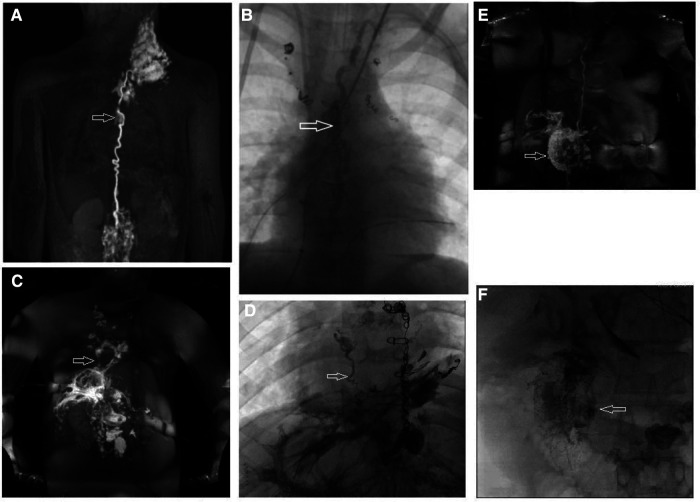
Different types of lymphatic interventions. (**B**) AP fluoroscopic imaging after thoracic duct embolization (arrow) in a 5 year old patient. (**A**) Corresponding preinterventional IN-DCMRL. (**C**) MIP coronal projection of IH-DCMRL in a 12 year old patient with plastic bronchitis demonstrating hepatopulmonary connections (arrow). (**D**) AP fluoroscopic image showing the hepatopulmonary connection after selective embolization (arrow). (**E**) Coronal projection of intrahepatic dynamic contrast magnetic resonance lymphangiography in a 21 year old patient with PLE demonstrating leak into the duodenum. (**F**) Fluoroscopic imaging of the duodenum after periduodenal (arrow) lymphatic embolization in the same patient.

Conservative therapy alone is often insufficient in treating lymphatic disease in Fontan patients. Based on the current pathophysiologic understanding of lymphatic flow disorders a series of new minimally invasive and surgical techniques have been invented during the last decade with promising results. Lymphatic catheter-based interventions can now be provided to treat most lymphatic disease in specialized centers. They can be divided into two categories, those that are aimed to occlude abnormal lymphatic ducts and those that are meant to decompress the entire lymphatic system (25). Several techniques have been proposed to selectively occlude abnormal lymphatic channels using glue, coils, or a combination (46, 79). Thoracic duct embolization was first described (80). It involves the transabdominal cannulation of the thoracic duct with a 22–24 gauge spinal needle, the introduction of a microcatheter and administering of a lipiodol glue emulsion that occludes the central lymphatic system (Figures 11A, B). Alternatively the thoracic duct can be accessed retrogradely (81). Thoracic duct embolization was successfully performed in patients with postoperative chylothorax and plastic bronchitis but if possible it should be avoided in Fontan patients because it can lead to the development of downstream lymphatic complications like protein loosing enteropathy or ascites (52, 80, 82). Nowadays a more selective approach is preferred whenever possible (Figures 11C, D) (33, 52, 83, 84). During selective lymphatic duct embolization procedures the microcatheter in the thoracic duct is further advanced into side branches or directly into small lymphatic fistulas that can subsequently be embolized. Sometimes a second microcatheter is placed in the thoracic duct and flushed with glucose during glue administration to prevent carryover of glue into the thoracic duct. In protein losing enteropathy patients abnormal lymphatic networks can selectively be punctured in the liver or the intestinal wall with a 25-gauge spinal needle with a similar approach used in liver lymphatic imaging (Figures 11E, F) (85). After endoscopic confirmation of an abnormal connection to the duodenum with blue dye these channels can then be sealed with glue. Another minimally invasive procedure that occludes lymphatic flow is ethiodized oil embolization. Lipiodol is an iodinated contrast agent that leads to sclerosis in small lymph vessels. It can be administered through a microcatheter or *via* needles placed in the inguinal lymph nodes. The latter strategy had been demonstrated to be successful in the treatment of chylothorax (86–88).

**Figure 12:**
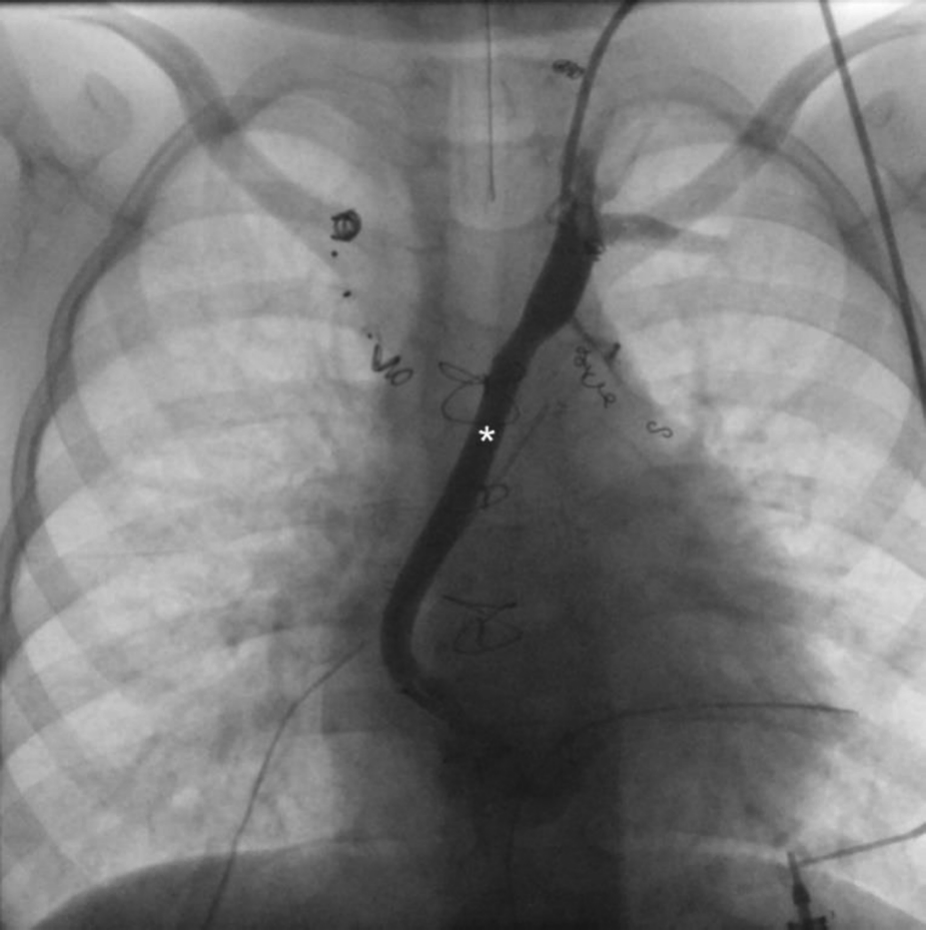
Ap fluoroscopic imaging of a 5 year old patient after surgical TD decompression demonstrating an unobstructed shunt (asterixis) between the innominate vein and the atrium. TD, thoracic duct.

A new therapeutic approach is the decompression of the lymphatic system. This can either be achieved surgically (Figure 12) or minimally invasively with catheter-based interventions (89–93). The goal is to redirect lymphatic flow that normally enters the innominate vein into the low-pressure environment of the atrium and thereby improving lymphatic drainage and preload. A concern in all these procedures is the iatrogenic creation of a right to left shunt. Many patients tolerate these shunts well but profound desaturation can occur. In such cases hypoxemia can be improved with banding of the internal jugular vein (94). For both minimally invasive and surgical decompression procedures an unobstructed thoracic duct outflow and a low atrial pressure levels are essential for therapeutic success and have to be evaluated before the procedure.

In patients where the thoracic duct outflow is obstructed novel microsurgical techniques like thoracic duct-to-vein-anastomosis may be beneficial (95, 96). In children with therapy refractory chylothorax the creation of a lympho-venous anastomosis has recently been described to be successful (95, 97). However, these case reports did not provide long term outcome data on how long the anastomosis stayed patent. In the past this was a great issue and has to be investigated carefully in the future.

Other surgical procedures in the management of lymphatic insufficiency that are less frequently used in the modern area include thoracic duct ligation, fenestration creation, Fontan takedown and pleurodesis (1, 38, 98).

In patients with ascites paracentesis can be diagnostic and therapeutic. A small number of case reports suggest that recurrent high volume ascites may affect Fontan hemodynamics adversely and that paracentesis may decline systemic venous pressure and increase cardiac output (1, 25, 99, 100).

In multicompartment lymphatic failure or severe therapy refractory protein losing enteropathy and plastic bronchits orthotopic heart transplantation can resolve symptoms (101, 102). In a recent meta-analysis the immediate survival rate of patients with Fontan failure was 88% and 61% after 10 years (103). Chronic kidney disease and two or more failures were associated with a higher risk of death in this analysis. Especially patients with severe lymphatic insufficiency are at increased risk of end organ damage that may limit their ability to be considered for transplantation (4). Together with the small number of suitable organs this strategy is not available for all patients. Early consideration and evaluation in a specialized center may improve quality of life and long time survival in these patients.

## Conclusion

The lymphatic system plays a central role in the development of some of the most devastating diseases associated with hypoplastic left heart syndrome. Lymphatic anatomy and function can now easily be assessed with novel imaging modalities in all major lymphatic compartments. Based on the recent pathophysiologic understanding new minimally invasive interventional procedures have been invented to target abnormal lymphatic channels and improve abnormal lymphatic flow in these patients with promising results. However, the new techniques are not widely available and evidence is limited to case series and expert opinions and long-term outcome has to be defined. There is a great need to further improve our understanding on what treatment strategy is best for which patient, who is at risk to develop lymphatic insufficiency and how to prevent lymphatic flow disorders in these prone individuals. The management of lymphatic failure still remains a challenge in the Fontan patient but there are now new prevailing therapeutic strategies available that can change outcome in the Fontan population.
